# Rejuvenated Stem/Progenitor Cells for Cartilage Repair Using the Pluripotent Stem Cell Technology

**DOI:** 10.3390/bioengineering8040046

**Published:** 2021-04-10

**Authors:** Naoki Nakayama, Sudheer Ravuri, Johnny Huard

**Affiliations:** 1 Steadman Philippon Research Institute, Fort Collins, CO 80526, USA; 2 Steadman Philippon Research Institute, Vail, CO 81657, USA; sravuri@sprivail.org

**Keywords:** pluripotent stem cell, cartilage, regeneration

## Abstract

It is widely accepted that chondral defects in articular cartilage of adult joints are never repaired spontaneously, which is considered to be one of the major causes of age-related degenerative joint disorders, such as osteoarthritis. Since mobilization of subchondral bone (marrow) cells and addition of chondrocytes or mesenchymal stromal cells into full-thickness defects show some degrees of repair, the lack of self-repair activity in adult articular cartilage can be attributed to lack of reparative cells in adult joints. In contrast, during a fetal or embryonic stage, joint articular cartilage has a scar-less repair activity, suggesting that embryonic joints may contain cells responsible for such activity, which can be chondrocytes, chondroprogenitors, or other cell types such as skeletal stem cells. In this respect, the tendency of pluripotent stem cells (PSCs) to give rise to cells of embryonic characteristics will provide opportunity, especially for humans, to obtain cells carrying similar cartilage self-repair activity. Making use of PSC-derived cells for cartilage repair is still in a basic or preclinical research phase. This review will provide brief overviews on how human PSCs have been used for cartilage repair studies.

## 1. Background: Pros and Cons of Adult Chondrocyte- and Adult Stem Cell-Based Cartilage Repair

Joint articular cartilage lacks spontaneous repair activity in adult humans and large animals [1,2], which can be attributed to lack of proper reparative cells and lack of environment for endogenous reparative cells to perform proper repair in the adult joint. The potential lack of endogenous reparative cells in the adult joint would be compensated by addition of chondrocytes or chondrogenic stem/progenitor cells into the joint space or directly to the injury site. In fact, many clinical approaches have been developed to repair joint cartilage injury by providing endogenous or exogenous reparative cells to the injury site. For example, the microfracture [3] and autologous matrix-induced chondrogenesis [4] methods rely on mobilization of endogenous cells, and the autologous chondrocyte implantation (ACI) [5] and matrix-associated ACI (MACI) [6] methods rely on the addition of ex vivo expanded chondrocytes and lately, mesenchymal stromal cells (MSCs). These surgical methods have been successfully applied to repair restricted types of injury, such as focal cartilage injury. However, methods to repair a variety of types of articular cartilage damage, including osteoarthritic wear-and-tear cartilage, which reproducibly achieve long-lasting regeneration of hyaline articular cartilage and prevention of progression to osteoarthritis, have not been developed yet [7,8]. Thus, joint injuries still bring many elderly and even young adults to the path to degenerative joint disorders such as osteoarthritis.

The most natural source of cells to stimulate repair of damaged articular cartilage is articular cartilage itself: i.e., articular chondrocytes. However, isolation of articular chondrocytes needs surgical isolation of healthy articular cartilage pieces from patients, which risks morbidity of the patients, followed by expansion of obtained chondrocytes in culture to yield a clinical scale of cell numbers, which rapidly loses their chondrocytic phenotypes. In contrast, MSCs are mesenchymal cells that can be isolated from a variety of tissues of patients, such as bone, bone marrow, fat, and synovial membrane and fluid [9,10]. They are commonly defined in vitro by their ability to adhere to and grow on plastic, and differentiate into chondrocytes, as well as other lineages such as adipocytes and osteoblasts (tri-lineage potential) under conditions optimized for individual lineages. MSCs are expandable at least for several passages to yield large numbers of them for therapeutic purposes before they lose the chondrogenic potential. Therefore, many MSC-based therapies for damaged articular cartilage, either by injuries or degenerative disorders, are currently investigated in clinical trials [10,11].

Not all MSCs show the same cartilage repair capacity [12]. MSCs from bone marrow and synovium seemed to be better for focal cartilage injury repair than MSCs from fat and muscle. MSCs from bone marrow (and periosteum) are considered to be originated from skeletal stem cells (SSCs) that are bone/bone marrow-resident multipotent skeletogenic cells defined in a way similar for hematopoietic stem cells: i.e., by sub-fractionation of bone and bone marrow cells using fluorescence activated cell sorting (FACS) without expansion culture, and in vivo validation of fractionated cells (e.g., multi-lineage [bone, bone marrow stroma, cartilage, and fat] differentiation in kidney capsule), different from the way MSCs is defined: i.e., expansion culture followed by in vitro validation, as described above [13,14,15,16]. Synovial MSCs are implicated in cartilage homeostasis [17]: e.g., increase in their number in synovial fluid in response to joint destabilization and cartilage degeneration [18,19]. Recently, synovium-resident multipotent skeletogenic cells have also been defined by FACS without expansion culture, although biological validation was performed in vitro [20]. Thus, MSCs from bone marrow and synovium appear to be relevant to bone and cartilage homeostasis [16], but thus far no clear differences between synovial MSCs and bone marrow MSCs in their capacity to form articular-like permanent cartilage and repair focal cartilage injury have been reported [12,21]. Furthermore, the endogenous cartilage reparative cells mobilized by microfracture from the subchondral bone (marrow) are likely to be SSCs [22]. However, as indicated by the repair outcome of microfracture treatment, the repair tissues induced by such endogenous SSCs are generally fibrotic [22], which do not last long, since their physical property is different from that of normal articular cartilage surrounding the repair site. ACI/MACI-like treatment using bone marrow MSCs also tend to result in a similar repair outcome [23,24]. Therefore, no clear differences between bone marrow MSCs and SSCs in their capacity to form articular-like permanent cartilage and repair focal cartilage injury have been noted, either.

## 2. Biologics for Improving Local Milieu for Endogenous and Exogenous Chondrogenic Cells to Properly Repair Articular Cartilage Damages

One of the strategies toward improving the outcome of exogenous or endogenous reparative cell-based cartilage repair is to optimize the local milieu for such cells to properly rebuild the articular cartilaginous tissue in the damaged site, using factors that are known to directly or indirectly enhance chondrogenesis from MSCs and SSCs in vitro and in vivo. Such factors include transforming growth factor beta (TGF-β) [25,26,27], bone morphogenetic protein (BMP) [28,29,30,31], fibroblast growth factor 18 (FGF18) [32,33], insulin-like growth factor 1 (IGF1) [34,35], and stromal cell-derived factor 1 (SDF1/CXCL12) [36,37]. These factors have been preclinically and clinically tested and resulted in some positive outcomes [8]. However, they also show negative effects on repair outcomes, presumably when they are overexpressed. For example, TGF-β stimulates joint fibrosis [38], BMP induces ectopic ossification, hypertrophic differentiation of repair chondrocytes, and osteophyte formation [38,39], and SDF1 recruits inflammatory cells and is involved in osteoarthritic degeneration of cartilage [40]. Actually, microfracture in a TGF-β-inhibitory environment, created by losartan treatment, showed significant improvement in the repair outcome in rabbits: i.e., hyaline cartilage regeneration [41]. These observations imply that these factors may need to be provided at a proper timing and dose in the vicinity of repair site.

In addition, it has recently been reported that the local milieu suitable for microfracture-activated SSCs to regenerate hyaline cartilage tissue within osteochondral defects is a combination of the anti-angiogenic factor, sFLT1 (soluble form of the vascular endothelial growth factor [VEGF] receptor 1) and BMP2 [22]. The beneficial effect of sFLT1 on stem cell-based hyaline cartilage repair of full-thickness defects was first demonstrated using muscle-derived stem cells (MDSC) in the presence of BMP4 in rats [42,43]. Administration of a neutralizing monoclonal antibody against VEGF alone also resulted in repair of full-thickness injury of articular cartilage with a hyaline cartilage tissue [44], and slowed the degradation of anterior crucial ligament-transected joint cartilage [45] in rabbits. Furthermore, an anti-angiogenic environment controls the direction of differentiation of SSCs. For example, sFLT1 converted SSC’s fate from bony tissue formation to cartilaginous tissue formation in a kidney capsule [14,15], and sFLT1-dependent induction of spontaneous chondrogenesis was noted in MSCs and in adipose tissue [14,46] in mice. However, considering that side effects are anticipated by systemic anti-VEGF treatments [47], and that transient presence of endothelial cells specifically in the mesenchymal condensation stage facilitates subsequent chondrogenesis [48], the anti-angiogenic environment may need to be provided locally at a proper timing during the repair process [49].

Thus, attempts to manipulate local milieu for the endogenous or exogenous therapeutic cells using known biologics have led to significant benefits in regeneration of hyaline cartilage in repair sites, which are however mostly based on relatively short-term (4–12 weeks) observations. Therefore, some of the regenerated hyaline cartilaginous tissues may not be permanent cartilage in that in a longer term, chondrocytes in the repair site may die or be committed to endochondral ossification: i.e., hypertrophic differentiation, terminal maturation and mineralization, leading to the degradation of repair tissue [50] and the formation of osteophytes [51]. Interestingly, death of superficial chondrocytes protects articular cartilage from joint destabilization-induced degradation [52], but loss of maturation arrest in living articular chondrocytes is associated with the onset of osteoarthritis [53]. In this respect, use of parathyroid hormone-related peptide (PTHrP), preferred ligands for the FGF receptor 3 (FGFR3), such as FGF9 and FGF18, and sFLT1 may have a beneficial effect on the maturation arrest of articular chondrocytes, since PTHrP [54,55,56] and FGF18 [57] are expressed in articular cartilage, which are known to block chondrocyte hypertrophic differentiation [58], and VEGF that sFLT1 targets is essential for chondrocytes to terminally mature and mineralize [49,59,60]. These factors have been tested for their roles on protection of articular cartilage from osteoarthritic degeneration [45,57,61,62], as well as on repair of damaged articular cartilage [22,42,43,44,63,64,65], but their effects on permanent repair has not been demonstrated. Much improvements have also been made to scaffold or hydrogel technology to provide a suitable micromilieu, in which embedded MSCs survive and differentiate [66], but optimization has been aimed mostly at enhancing chondrogenesis, rather than at preventing terminal maturation, mineralization, and degradation of the resultant chondrocytes [67].

Thus, robust, biologics-induced chondrogenesis in vivo may be helpful in inducing hyaline cartilage repair tissue, instead of fibrotic repair tissue [68,69,70], but further optimization of the therapeutic strategy to develop articular-type permanent cartilage, for example by providing suppressive environment for chondrocyte maturation, has not been extensively investigated. It is worth mentioning that more extensive searches for therapeutic molecules, which involve comparative genetic, genomic, epigenomic and proteomic analyses of intrajoint tissues such as articular cartilage, ligament, synovium, and synovial fluid [71], have nominated a number of genes and proteins expressed in the intrajoint tissues in a disease condition-dependent manner. Validating effects of such genes and proteins on cartilage repair is still ongoing.

## 3. Potential Advantage of Embryonic Chondroprogenitors, as an Alternative Cell Type for Cartilage Repair

**Figure 1:**
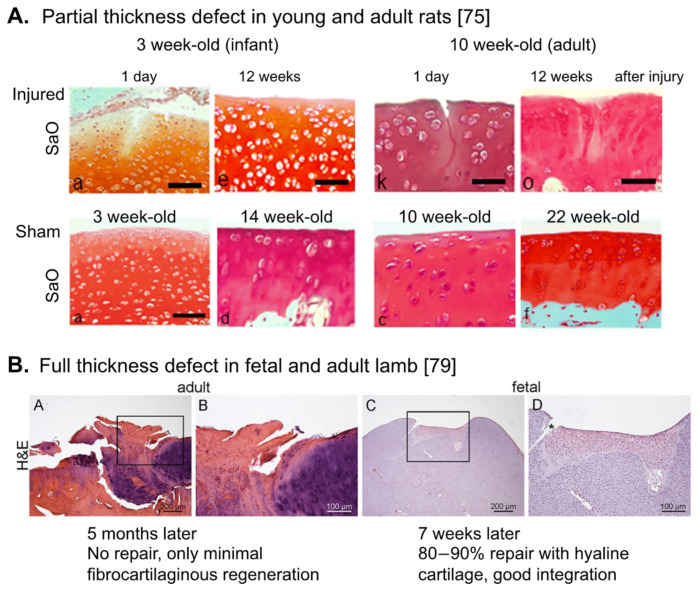
Repair activity in the fetal, young and adult articular cartilage. Histological changes of articular cartilage after introducing partial-thickness injury (**A**) and full-thickness injury (**B**) in the fetal (**B**), young/infant (**A**), and adult (**A**,**B**) animals. (**A**) Taken from Akatsu, et al. [75]. Note that hyaline cartilage repair with good integration was observed in 3 week-old infant rats. (**B**) Taken from Ribitsch, et al. [79]. SaO: Safranin O, H&E: Haematoxylin-Eosin staining.

Another strategy to improve the outcome of cell-based cartilage repair is to take the age of reparative cells into account. In contrast to adult joint cartilage, embryonic/fetal joint cartilage possesses spontaneous scar-less repair activity for a chondral (partial-thickness) defect [72]. In small animals, such activity continues to the postnatal infant stage. For example, 3 week-old infant rats [73,74,75], and 12–14 week-old young rabbits [76,77,78] spontaneously repair partial-thickness defects of joint articular cartilage, but these activities disappear in the adulthood. Full-thickness and osteochondral defects show some spontaneous repair activity even in adult animals, but repair tissues are normally fibrotic, similar to the repair outcome of the microfracture method [3,22]. However, still the younger the better, since full-thickness and osteochondral defects in fetal [79] (Figure 1B) and adolescent animals (e.g., 6 week-old rats and 13–15 week-old rabbits) [77,78,80,81,82], show faster and hyaline cartilage repair, dependent on the size of injury.

During embryogenesis, the entire synovial joint, including articular cartilage, ligament and synovium, develops de novo from a specific group of progenitor cells called interzone cells or joint progenitors, that are marked by the Growth and Differentiation Factor 5 (*Gdf5*) transcript [83,84]. These cells are distinct from the Sry-box *(Sox) 9*-expressing “general” chondroprogenitors [85], destined to primarily generate growth plate chondrocytes. GDF5 is a member of the BMP family, expressed in articular chondrocytes and needed for maintenance of articular cartilage [86,87,88,89]. Interestingly, the mouse *Lgr5^+^Gdf5^+^* interzone cells facilitate repair of osteochondral defects of adult articular cartilage [90]. Recently, genetic lineage tracing experiments have demonstrated that progeny of the embryonic *Gdf5*^+^ interzone cells (‘*Gdf5*-lineage’ cells) remain in the synovium of the adult mouse joint, that synovial MSCs are mostly originated from such *Gdf5*-lineage cells, and that osteochondral defects of articular cartilage stimulate proliferation of and chondrogenesis from the *Gdf5*-lineage cells, and recruit them to the defect site [91]. These observations suggest that the cartilage self-repairing activity in the joint of embryonic and infant rodents and rabbits may be elicited by the synovial *Gdf5*-lineage cells.

Similar lineage tracing experiments have also demonstrated that *Prg4* (*Lubricin*)^+^ cells of embryonic (E14.5) to newborn (P0-5) mouse articular cartilage appear to represent the articular cartilage stem/progenitor cells enriched in the superficial zone of articular cartilage [92], and contribute to articular cartilage growth by populating chondrocytes in all layers of articular cartilage till the infant stage in mice [93,94,95]. Therefore, *Prg4*^+^ embryonic and infant superficial zone chondrocytes can also contribute to the cartilage self-repairing activity in embryonic and infant joints, and may be able to “repopulate” articular chondrocytes in adult articular cartilage if provided exogenously.

While injury-induced micromilieu for endogenous reparative cells in embryonic and infant joints may be different from that in adult joints, and may play an important role on the spontaneous repair of articular cartilage defects, these observations suggest that SSCs [13,14,15,22,96], synovial *Gdf5*-lineage cells [91], *Prg4*^+^ superficial zone chondrocytes [92,93,94,95], and *Gdf5*^+^ interzone/joint progenitor cells [90] in embryonic and infant joints: i.e., young reparative cells, may be a better alternative to adult MSCs and SSCs to achieve the therapeutic goal: i.e., repair of articular cartilage injury with long-lasting hyaline cartilage tissues leading to prevention of injured cartilage from progressing to osteoarthritis.

## 4. Pluripotent Stem Cells as a Source for Embryonic Articular Chondroprogenitors for Humans

Thus, the embryonic and infant chondroprogenitors and chondrocytes may be interesting cell-types to test for their capability of long-term hyaline cartilage repair in adult articular cartilage, but these cells are not easily obtained from humans in a clinical quantity. In contrast, pluripotent stem cells (PSC) such as embryonic stem cells (ESC) and induced PSCs (iPSC) are capable of differentiating into all somatic cells, through processes that mimic early embryogenesis, and the resulting cells tend to carry embryonic characteristics. PSCs can be expanded in culture almost indefinitely, too. Therefore, for humans, PSCs are the only practical source for obtaining large numbers of embryonic/fetal cell-types. Methods to generate embryonic chondrocytes as well as embryonic chondroprogenitors from mouse (m) and human (h)PSCs have been established by many groups, which have been reviewed previously [97,98]. We have also previously established and refined signaling requirements for the differentiation of PSCs to three embryonic precursors of chondrocytes, namely lateral plate mesoderm, paraxial mesoderm, and (cranial) neural crest [99,100,101,102,103,104,105,106,107]. Interestingly, we and others have also shown that hPSC-derived chondrogenic cells of the mesodermal origin gave rise to hyaline cartilage pellets in vitro [104], which were maintained to some extent as an unmineralized state in vivo, especially when BMP signaling was limited in a late stage of the in vitro chondrogenesis culture [106,108,109]. These observations suggest that PSC-derived chondrogenic mesodermal cells may contain progeny that are committed to generate permanent chondrocytes: i.e., chondrocytes that resist endochondral ossification, the process which stimulates chondrocyte hypertrophy, terminal maturation, and mineralization to form bone as in the growth plate.

The PSC-derived chondrogenic mesenchymal cells can be expanded in a serum-containing medium or in a specialized serum-free medium (e.g., FGF2 + TGF-β receptor inhibitor) [98]. When expanded under the serum-free condition, hPSC-derived chondroprogenitors well maintain their hyaline chondrogenic activity for over 15 passages [106], but chondrocytes developed from such expanded cells acquire tendency to commit themselves to the endochondral ossification process: i.e., cartilage pellets developed with them express signs of hypertrophic differentiation (e.g., transcripts of the type X collagen and alkaline phosphatase genes) in vitro and readily form a bony tissue in vivo, similar to adult MSC-derived cartilage pellets [70,110,111]. These observation suggest that chondroprogenitors, generated in culture from mesodermal progeny of hPSCs and expanded in a way to maintain long-term their hyaline chondrogenic activity, somehow lose their capacity to form permanent chondrocytes.

## 5. Development and Isolation of Chondrogenic Cells from Pluripotent Stem Cells

There is a report that full-thickness defects of sheep articular cartilage were successfully repaired by providing undifferentiated sheep ES-like cells in a fibrin glue [112]. However, PSCs are tumorigenic, i.e., teratoma-forming cells, and the teratoma-forming activity has been the definition of pluripotency for hPSCs [113,114,115]. Therefore, lineage-restricted progenitor cells differentiated from PSCs are considered more suitable for therapeutic purposes than PSC themselves, but risk of contamination of tumor forming, undifferentiated PSCs in the differentiated PSC population remains [116,117]. In fact, when hPSCs, especially hiPSCs, are differentiated into chondrocytes or chondroprogenitors that are used without a step to purify them or their precursors by physical methods: e.g., FACS and magnetic-activated cell sorting (MACS), or by biological methods: e.g., expanding specifically the differentiated cell-type of interest in culture, immature teratoma-like tumor is developed in cartilage mass generated from them in vitro [118], and in an immunodeficient mouse knee after transplantation of them for 16 weeks [119]. Therefore, the safest way to regenerate cartilage using hPSCs is to include a step in the protocol to physically or biologically eliminate the tumor forming, undifferentiated PSCs, prior to transplantation.

In early studies, biological methods: e.g., selective expansion culture, were mainly employed for enriching or purifying chondrogenic mesenchymal cells or MSCs [98]. PSCs were differentiated by way of forming embryoid bodies (EB) in vitro. Then, mesenchymal cells growing out of EBs, called EB outgrowth cells, were selectively expanded in media similar to those developed for expanding bone marrow MSCs, prior to induction of chondrogenesis and use for cartilage repair analyses [98].

**Figure 2:**
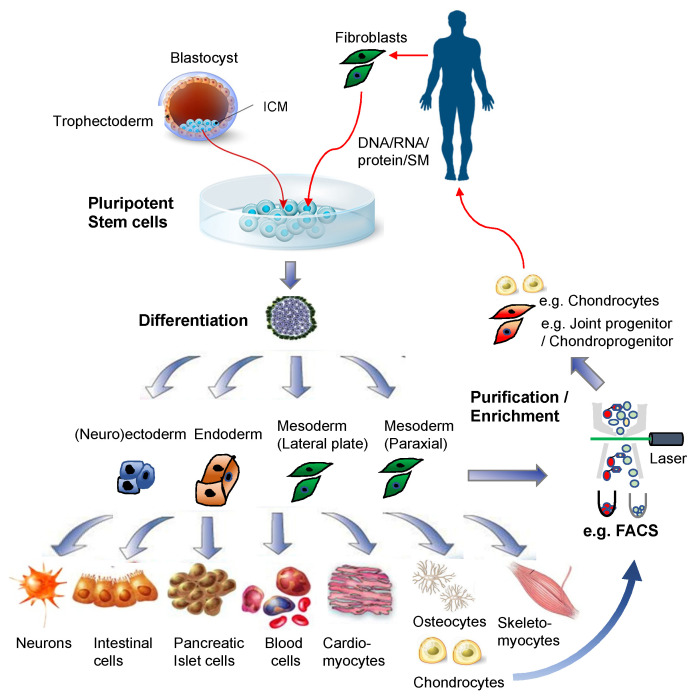
Pluripotent stem cells for regenerative medicine. Illustrations for the blastocyst, pluripotent stem cells, and differentiated cells were purchased from Dreamstime.com.

Recent studies tend to make use of antibody-based physical separation methods to enrich PSC-derived chondroprogenitor cells or their precursors such as PSC-derived mesoderm or neural crest (Figure 2). For example, FACS-isolated VEGF receptor 2 (FLK1/KDR)^−^ platelet-derived growth factor receptor alpha (PDGFRα)^+^ EB cells are chondrogenic mesodermal progeny of PSCs [99,100,104,105]. The hPSC-derived mesodermal progeny, enriched by FACS-isolation of KDR^−^CD146^+^CD166^+^ BMP receptor 1B (BMPR1B)^−/lo^ cells, or by MACS-depletion of contaminated epithelial endodermal, cardiovascular, and hematoendothelial mesodermal cells, as well as undifferentiated hPSCs, are chondrogenic [120,121]. Furthermore, FACS-isolated green fluorescence protein (GFP)^+^ cells from the type II collagen gene *(Col2a1*) promoter-GFP knocked-in PSCs are enriched in chondrogenic progeny [122,123], and FACS/MACS-purified CD271^+^ hPSC-derived neural crest cells generate chondrogenic ectomesenchymal cells [106,124,125].

As for hPSC-derived MSCs, surface markers such as CD73, CD24, CD105, and CD90 have been used for detecting and isolating them by FACS, as reviewed in [98]. However, since MSCs can be relatively easily generated via spontaneous differentiation of hPSCs, and enriched by expansion culture in the standard, serum-containing MSC medium, FACS/MACS is not widely employed for purifying or enriching PSC-derived MSCs. However, the developmental process of mesodermal MSCs from hPSCs was first defined by Slukvin’s group using FACS isolation of mesodermal progeny [126]. Their method generates Apelin receptor^+^ mesoderm (that is PDGFRα^+^KDR^+^ and Lin^-^ [VE-cadherin^-^CD31^−^CD73^−^CD43^−^CD45^−^], and expresses *T*, *MIXL1,* and *FOXF1*: i.e., primitive streak and lateral plate mesoderm transcripts) from hPSCs, isolates them by MACS, and subjects them to mesenchymal colony forming culture to generate PDGFRβ^+^ CD271^+^Delta-like1(DLK1)^+^CD73^−^ primitive mesenchymal cells (expressing *PRRX1*: i.e., limb bud mesenchyme transcript). Then, PDGFRβ^+^CD73^+^CD90^+^ MSCs are generated from them in the presence of FGF2 in a serum-free medium [127].

## 6. Cartilage Tissue Engineering Using Pluripotent Stem Cell-Derived Chondroprogenitors

Use of PSC-derived chondrogenic cells for articular cartilage repair has not been extensively performed. Many early studies employed methods to generate chondrogenic mesenchymal cells or MSCs (e.g., EB outgrowth cells) from spontaneously differentiated PSCs, expand and prime them, and then use them for repairing damaged articular cartilage. Hwang et al. [128] and Toh et al. [129,130] have convincingly demonstrated, using this strategy, that hESC-derived EB outgrowth cells are capable of repairing damaged articular cartilage at least up to 12 weeks, when the cells were either embedded in a hyaluronan-hydrogel followed by pre-differentiated toward chondrocytes for 4 weeks in the presence of BMP7 and TGF-β1 [129,130], or expanded in chondrocyte-conditioned medium, followed by pellet cultured for 3 days [128], prior to transplantation. Similarly, Gibson, et al. has demonstrated that use of MSCs, which had been generated by a 2-dimensional, spontaneous differentiation method of hESCs and pellet cultured with BMP2 for 2 days and then with WNT5a for 12 days, showed statistically significant improvements in the repair of damaged articular cartilage [131]. In contrast, EB outgrowth cell-derived MSCs that had been complexed with poly(lactic-co-glycolide) scaffold and transplanted to full-thickness defects of rabbit articular cartilage without any pre-treatments, such as chondrogenic differentiation or chondrocyte-conditioned medium treatment, showed only a weak repair [132]. Effects of various biomaterials have also been explored but mostly in vitro, which have been reviewed elsewhere [103,133].

More refined lineage-restricted (e.g., mesodermal) chondrogenic mesenchymal cells were also used for cartilage repair. Ferguson, et al. [120] identified cell surface markers that can be used for identifying and isolating chondrocytes from different locations in human fetal articular cartilage. The integrin alpha 4 (IGTA4)^−^ BMPR1B^+^ chondrocytes that demonstrate the strongest matrix-depositing activity are form transitional zone, and the IGTA4^+^BMPR1B^+^ chondrocytes that show osteochondrogenic activity and PRG4 expression are from superficial zone. Interestingly, when mesodermal progeny of hPSCs generated based on the method of Wu, et al. [121] were purified by MACS-depletion of epithelial endodermal cells, cardiovascular and hematoendothelial mesodermal cells as well as undifferentiated hPSCs, and then differentiated by pellet culture for 60 days, the resulting cartilage pellets were enriched in IGTA4^+^BMPR1B^-^ mesenchymal cells, with a minor population of IGTA4^+^BMPR1B^+^ superficial chondrocytes. These cartilage pellets were capable of repairing a focal lesion of rat articular cartilage in as soon as 30 days [120].

Similarly, Gardner, et al. [134] reported hPSC-derived mesodermal cartilage tissue also repair a focal osteochondral defects of articular cartilage in nude rats. They employed Craft et al.’s method of mesodermal differentiation of hPSCs [108], followed by EB outgrowth cell generation and expansion for 12 days in a serum-free medium to get chondrogenic mesenchymal cells. Then, these cells were subjected to TGF-β3-based micro-mass culture for 12–15 weeks to generate cartilage mass that was used to fill the osteochondral defects. The quantitative analyses of repair outcome based on the ICRSII scoring system showed statistically significant improvement 12 weeks, but not 6 weeks after transplantation of the hPSC-derived cartilage mass.

The first demonstration of significant cartilage repair by hPSC-derived chondrogenic progeny, without pre-differentiation or chondrocyte-condition medium treatment prior to transplantation, was reported by Cheng et al. [135]. Their method gives rise to SOX9^+^ chondroprogenitors and chondrocytes via mesodermal progeny of hESCs, based on Oldershaw et al.’s 2-dimmensional hESC differentiation method [136] that has been improved to bring the SOX9^+^ cell population up from 75 to 95%, by removing the day-12 obligated split during chondrogenesis stage of differentiation culture. These SOX9^+^ cells encapsulated in fibrin glue resulted in better repair outcome than spontaneous repair of a focal osteochondral defect of articular cartilage in nude rats from 4 to 12 weeks [135].

More direct roles of PSC-derived chondroprogenitors or chondrocytes on repairing a damage of articular cartilage were demonstrated by organ culture systems. Diekman, et al. [123] showed that Col2a1-GFP^+^ cells isolated from differentiating mPSCs by FACS and embedded in 1% agarose were capable of regenerating cartilage matrices within a chondral defect introduced in pig explant cartilage in 21 days of culture. In addition, Wu, et al. [121] demonstrated that FACS-purified CD166^−/lo^ BMPR1B^+^ prechondrocytic cells, which had been generated by a 12–15-day chondrogenesis culture of CD166^+^CD146^+^KDR^−/lo^EpCAM^-^BMPR1B^−/lo^ hPSC-derived mesodermal cells in the presence of TGF-β1 and Leukemia Inhibitory Factor, contributed to repair defects introduced into a human fetal hip joint explant in 14 days of culture. These observations suggest the capacity of PSC-derived chondroprogenitors or chondrocytes to retain in defects sites of articular cartilage and regenerate cartilage matrices.

## 7. Conclusions and Future Prospective

PSC-derived MSCs, chondroprogenitors and chondrocytes have thus far given positive results in repairing focal full-thickness lesions in articular cartilage, using scaffold-free or scaffold/hydrogel-dependent methods, in small animal models and in organ culture models. However, their effects on age-related cartilage degenerative disorders such as osteoarthritis have not been extensively examined, while the beneficial effects of adult MSCs on osteoarthritis are now recognized, which is based on their trophic (e.g., anti-inflammatory) effects rather than their chondrogenic potential [10,137].

**Figure 3:**
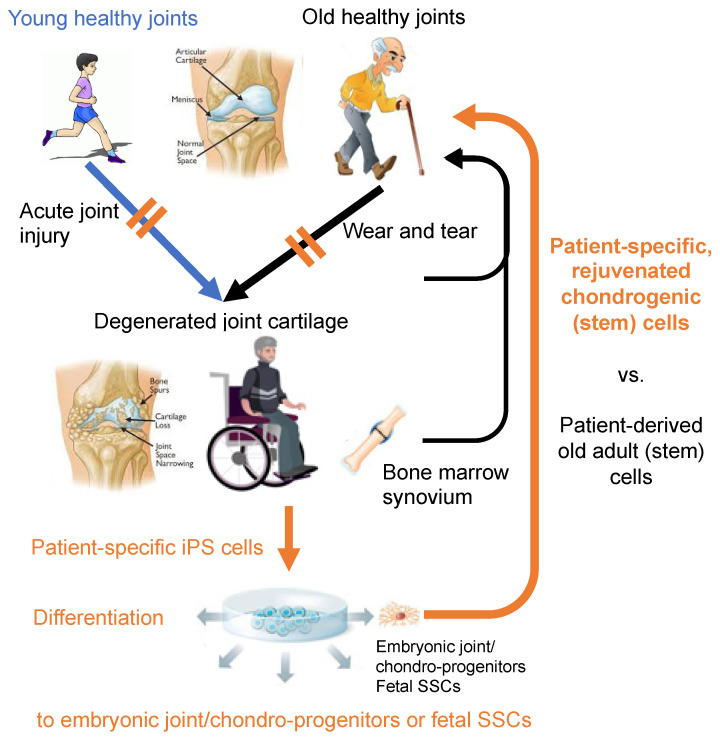
In total, two sources of patient-specific cells for regeneration of injured and degenerated cartilage: adult cells vs. rejuvenated (i.e., iPSC-derived) cells. Does rejuvenation make difference in the outcome of joint cartilage repair? The joint illustrations are in courtesy of OrthoInfo © American Academy of Orthopaedic Surgeons. Other illustrations were purchased from Dreamstime.com.

One of the major advantages of the iPSC technology on clinical application is to be able to get patient specific, “rejuvenated” cells [138,139,140,141] (Figure 3). Currently, it requires technically demanding two-step processes: i.e., gene/RNA/protein transfer or small molecule treatment to effectively reprogram adult cells to PSCs, followed by directed differentiation of the obtained PSCs to the cell-type of interest. In contrast, the “direct reprogramming” technology depends on one step process: i.e., gene/RNA/protein transfer, and is capable of inducing somatic cells, such as fibroblasts, to transdifferentiate to another type of somatic cells, such as neurons [142], osteoblasts [143], and chondrocytes [144], without going through the PSC stage. Therefore, this method will also eliminate the concern of contamination of teratoma-forming activity. However, such direct reprogramming technology seems to transfer the same problems or risks associated with the age of original somatic cells over to the reprogrammed cells [139,145]. The aging of adult stem cells has been noted, and attributed to the diminished regenerative activity in aged adult tissues [139,146,147]. Therefore, whenever rejuvenated stem cells are expected to show improved clinical outcome from a cell-based regenerative therapy, patient-specific hPSC-derived cells are likely suitable over cells isolated from patients or those directly reprogrammed. In this respect, while adult MSC-based therapies for injured or degenerated articular cartilage are currently being trialed [10,11], it would be of great interest in examining whether patient-specific hiPSC-derived embryonic MSCs and SSCs, or chondroprogenitors will result in better repair outcome than adult MSCs.
